# Rapid measurement of epidermal thickness in OCT images of skin

**DOI:** 10.1038/s41598-023-47051-6

**Published:** 2024-01-26

**Authors:** Chieh-Hsi Lin, Brandon E Lukas, Ali Rajabi-Estarabadi, Julia Rome May, Yanzhen Pang, Carolina Puyana, Maria Tsoukas, Kamran Avanaki

**Affiliations:** 1 Department of Computer Science, University of Illinois at Chicago, Chicago, IL 60607 USA; 2 Richard and Loan Hill Department of Bioengineering, University of Illinois at Chicago, Chicago, IL 60607 USA; 3 Dr. Phillip Frost Department of Dermatology and Cutaneous Surgery, University of Miami Miller School of Medicine, Miami, FL 33136 USA; 4 Department of Dermatology, Broward Health Medical Center, Fort Lauderdale, FL USA; 5 University of Illinois College of Medicine, Chicago, IL 60607 USA; 6 Department of Dermatology, University of Illinois at Chicago, Chicago, IL 60607 USA

**Keywords:** Skin cancer, Optical imaging

## Abstract

Epidermal thickness (ET) changes are associated with several skin diseases. To measure ET, segmentation of optical coherence tomography (OCT) images is essential; manual segmentation is very time-consuming and requires training and some understanding of how to interpret OCT images. Fast results are important in order to analyze ET over different regions of skin in rapid succession to complete a clinical examination and enable the physician to discuss results with the patient in real time. The well-known CNN-graph search (CNN-GS) methodology delivers highly accurate results, but at a high computational cost. Our objective was to build a computational core, based on CNN-GS, able to accurately segment OCT skin images in real time. We accomplished this by fine-tuning the hyperparameters, testing a range of speed-up algorithms including pruning and quantization, designing a novel pixel-skipping process, and implementing the final product with efficient use of core and threads on a multicore central processing unit (CPU). We name this product CNN-GS-skin. The method identifies two defined boundaries on OCT skin images in order to measure ET. We applied CNN-GS-skin to OCT skin images, taken from various body sites of 63 healthy individuals. Compared with CNN-GS, our described method reduced computation time by 130 $\times$ with minimal reduction in ET determination accuracy (from 96.38 to 94.67%).

## Introduction

Epidermal thickness (ET) changes are associated with several skin diseases such as Vitiligo^1^, diabetic foot^2^, psoriasis, atopic dermatitis, basal cell carcinoma, squamous cell carcinoma, melanoma, and many more^3–9^

The epidermis is the skin’s outermost layer that is a defense mechanism against a hostile environment. ET can be measured non-invasively using identified skin layer boundaries on images acquired by optical coherence tomography (OCT). Traditional OCT analysis manually extracts the layer border positions and subsequent structural characteristics, which can be laborious, time-consuming, and prone to errors. Hence, there is great value in an automatic, efficient, and accurate segmentation method to reduce errors and minimize the need for training on interpretation of OCT images. When OCT is used in the clinic, the handheld device is used on the skin and moved to different parts of lesions to detect potential abnormalities. A fast analysis of skin layers is essential to complete a clinical examination and enable the physician to discuss results with the patient in real time.

Neural network models are commonly adopted to assist in this type of image analysis. Such methods include convolutional neural networks (CNN)^10^ and recurrent neural networks (RNN)^11^. Both of these neural frameworks have been applied in diverse medical image analysis, such as brain tumor segmentation^12^, retinal blood vessel segmentation^13^, retinal lesion detection^14^, and skin segmentation^15–17^. A further refinement, which has shown increased accuracy, is the use of a hybrid structure combining a CNN model with two or more approaches. Chen et al.^18^ worked on semantic image segmentation using a deep CNN model with fully connected conditional random fields. Gophinath et al.^19^ utilized a CNN model for extracting layers of interest and edges from the images, while, simultaneously, bidirectional long short-term memory (BLSTM) tracing the layer boundaries on segments of retinal layers. Fang et al.^20^ applied this patch-wise based CNN model with a graph search algorithm (CNN-GS) to segment retinal layer boundaries in OCT images of non-exudative age-related macular degeneration (AMD). Likewise, Kugelman et al.^21^ attempted the same composition but used the RNN model (RNN-GS) instead to segment retinal layers from healthy children and adult patients with AMD, which showed comparable results to the CNN-GS model. While CNN-GS and RNN-GS have demonstrated excellent accuracy for analyzing retinal layer boundaries, we have found they are somewhat computationally expensive to implement for OCT skin image analysis. Some of the results contained herein were presented previously in a brief conference paper^22^, but due to considerable positive feedback, we here present a more thorough elulcidation of the method developed and additional results. Here we have thoroughly explained the testing of speed-up algorithms, optimization of hyperparameters, selection of appropriate loss function, and, most significantly, use of our novel pixel-skipping algorithm.

Below we describe a comprehensive modification of CNN-GS, which we dub CNN-GS-skin, and how it can be used to detect boundaries of the epidermal layer including stratum corneum (SC) and dermal-epidermal junction (DEJ), and in this way, measure ET. We aim to develop a computation-efficient method while preserving reasonable accuracy on ET measurement. We then compare CNN-GS-skin to CNN-GS in terms of accuracy, number of parameters required to be computed, and overall execution time. We also describe an implementation of skin segmentation on a widely-used deep learning image analysis method, UNet. Finally, we compare position accuracy and ET thickness for different body sites measurements relative to manual segmentation for CNN-GS-skin, CNN-GS, and UNet.

## Methodology

### Imaging system

W used a multi-beam, swept-source OCT (SS-OCT) system (Vivosight, Michelson Diagnostic Inc.). The OCT system is an FDA-approved machine with a hand-held scanning probe for skin imaging. The lateral and axial resolutions are 7.5 $\upmu$m and 10 $\upmu$m, respectively. The scan area of the OCT system is 6 mm (width) $\times$ 6 mm (length) $\times$ 2 mm (depth). A tunable broadband laser source (Santec HSL-2000-11-MDL), with central wavelength of 1305 $\nu$m, successively sweeps through the optical spectrum and leads the light to four separate interferometers and forms four consecutive confocal gates.

### Dataset description

All of the imaging procedures and experimental protocols were approved and carried out according to the guidelines of the University of Illinois at Chicago’s Institutional Review Board (IRB). To implement the proposed method comprehensively and to ensure a diverse set of features, we created a large dataset of OCT images composed of 315 stacks of OCT images taken from the forearm, back of hand, forehead, neck, and palm of 63 healthy individuals. Each stack has 50 images from adjacent transverse locations, creating volume data. We used 5 images from each stack for processing. Each image is 460 $\times$ 1500 pixels (1.5 mm $\times$ 6 mm). Our dataset included a total of 1575 images, 70% (selected randomly and divided between the five body sites evenly) were used for CNN model training, and the remaining images were used for testing and validation. To prepare the images for model training and testing, each OCT image was manually annotated with two-layer boundaries, labeled Boundary 1 for SC, the upper bound of the epidermis; and Boundary 2 for DEJ, the boundary that separates epidermis and dermis. For each OCT image, manual segmentation was done blindly by 4 independent medical professionals (two medical students and two dermatology fellows), who all had been trained in OCT image segmentation and OCT image interpretation and have experience in OCT interpretation. We used the median location between the boundaries blindly performed by the 4 independent markers as the ground truth to train and test the models. In doing this, we observed a maximum deviation of 4 pixels, minimum deviation of zero pixels and an average deviation of 2 pixels for all of the images. Segments were delineated using pixel-wise labeling, where each boundary pixel was assigned a label (pixels are 4 (width) $\upmu$m $\times$ 3.26 (depth) $\upmu$m.

**Figure 1:**
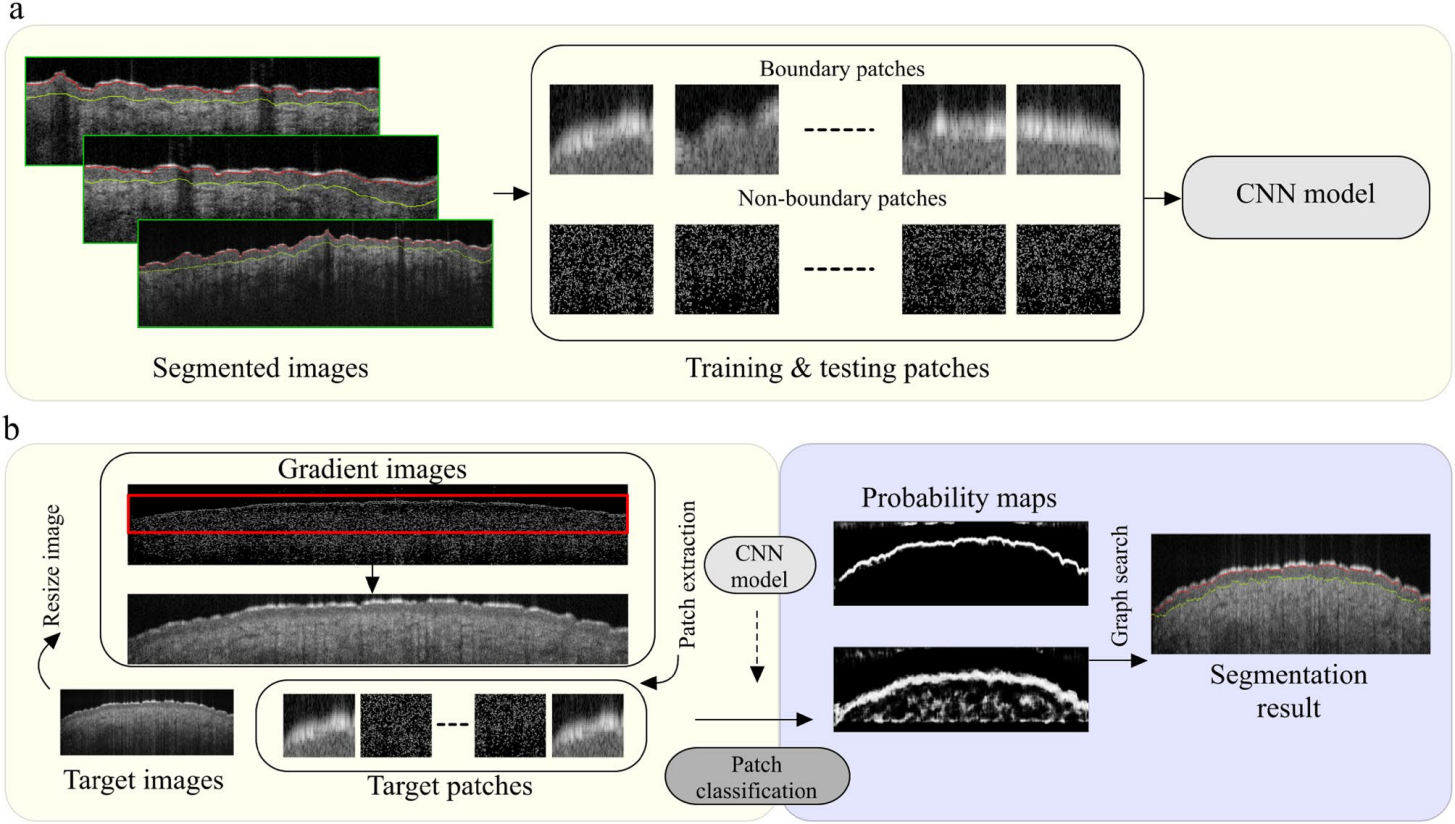
Overview of CNN-GS framework. (**a**) Training CNN model. (**b**) Evaluating CNN-GS segmentation. Yellow block: stage 1—CNN-based patch training and classification (interior red block is the area selected to look for boundaries); blue block: stage 2—probability maps and graph search. Image courtesy of^22^.

### Proposed framework

We implemented three different frameworks for automated ET thickness detection to analyze the OCT images to identify the boundaries. The first one, following Fang^20^, was a combination of a patch-wise based CNN model and a graph search algorithm (CNN-GS). The second framework, CNN-GS-skin, modifies the parameters from *Fang* and implements additional compression techniques to reduce computational time without significant loss of accuracy. The third framework (for comparison of computational time and accuracy) was an implementation of UNet described below.

The rationale for the use of a CNN-GS is as follows: CNNs are more effective when looking at smaller sections of an image to find specific features. The use of small sections also leverages the fact that nearby pixels are more strongly related than distant ones, as evaluated by traditional neural networks^23^. Graph Search is an algorithm that systematically explores all the vertices and edges of a graph: In *Fang*, as in our method, Dijkstra’s algorithm is used to implement graph search. In Chiu et al.^24^, for example, an OCT image is taken as the graph where each pixel represents the vertex of the graph, and neighboring pixels are connected by edges. Each connected edge is assigned a value of the weight, termed edge-weight. The edge-weight is a user-defined value depending on the users’ requirements. The shortest path found by the algorithm is a set of connected edges with a minimum cost of edge-weight. The framework is constructed by Keras API with Tensorflow^25^. We implemented CNN-GS in Python. CNN-GS contains two stages: (1) patch-based CNN training and classification; and (2) probability maps and graph search. Figure 1 illustrates the outline of a CNN-GS framework; and the color blocks represent the two stages.

#### Stage 1: Patch-based CNN training and classification

Prior to the training phase, we normalized the images in the range between 0 and 1^26^. To train the CNN model as a patch-based classifier, we created a dictionary of patches from and corresponding labels as the input to train the model. These patches are overlapped patches of size 55$\times$55 pixels extracted from the normalized training images. Each patch is assigned a class label based on the center pixel. The patches are labeled 1 and 2 according to their layer boundaries and assigned label 0 for those that are not centered on the layer border. The patchset was balanced by randomly selecting the same amount of boundary and non-boundary patches from all patches. Both patches and patch labels are used to train the CNN model. Once the training process is completed, the CNN model is optimized by fine-tuning hyperparameters.

**Figure 2:**
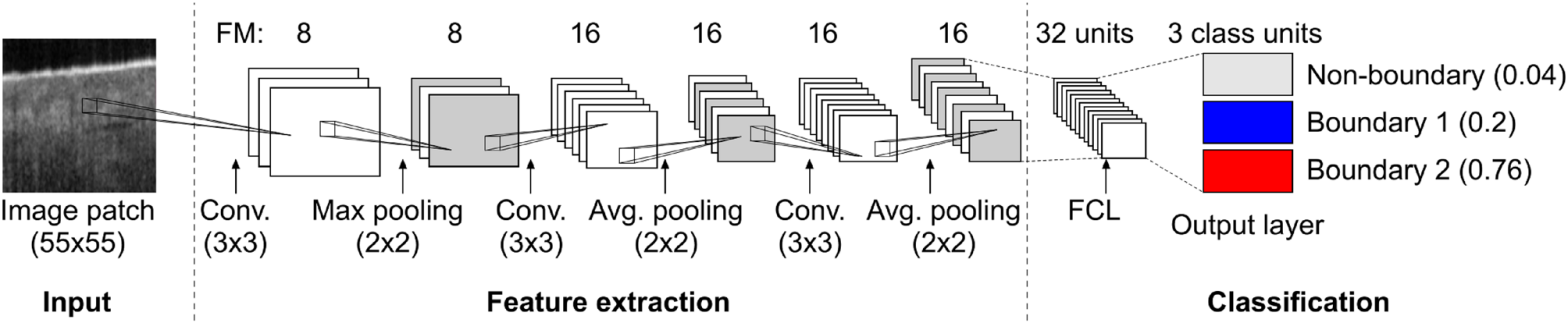
CNN model using method described in^20^ with convolution layers, pooling layers, and fully connected layers. *FM* feature maps, *FCL* fully connected layer. Image courtesy of^22^.

CNN-GS, as implemented by Fang et al.^20^, is not particularly efficient for fast OCT skin image analysis. For a more practical implementation of CNN-GS, we aimed to develop a model which spends less computation time on execution, regardless of the training time, and can be implemented in a multicore central processing unit while still preserving satisfactory classification outcomes. With these conditions in mind, we first examined the impact of various hyperparameters to speed up execution times. Figure 2 shows CNN-GS, containing three blocks of convolutional and pooling layers, and a fully connected block consisting of hidden layers and an output layer.

To segment OCT images for training the CNN model, that is, to create the training patchsets, we first deleted the region on top of the OCT image, above the SC, which is the image of air. We detected the edge pixels generated from the gradient of the image due to the great contrast between the pixel intensities of air and SC; and utilized non-local means filtering to reduce speckle^27^. The above pre-processing assists in finding the approximate outermost layer boundary to constrain the target predicting area. To lessen the blunder from the edge detection procedure, from the outermost boundary found, we extended a few rows of pixels above it and fixed the row size beneath it to create a smaller target image, decreasing the target patchset size and the computation time for classifying. The training patchset is then created from the modified target image. The output of the CNN model is three probability maps, corresponding to three defined labels, and a class label that has the highest probability of showing which class the patch will belong to. These predicted probability maps are required for *Stage 2*.

#### Stage 2: probability maps and graph search

Since an OCT image may consist of several layer boundaries, when segmenting on a specific boundary, users are required to select or estimate corresponding boundary start and end nodes for each per-class probability map constructed from the output of the machine learning model. Therefore, before directly creating the graph from the probability map of the target image, we implemented automatic endpoint initialization^24^, which is a method that bypasses the need for manual end-point selection. This initialization is based on two assumptions: (1) the layer segmentation extends across the entire width of the image; and, 2) Dijkstra’s algorithm preferentially finds the minimum weight of the path. The edge-weight of the connected edges, $w_{cn}$ is given by the equation:

1 $$\begin{aligned} w_{cn} = 2-(P_{c}+P_{n})+w_{min} \end{aligned}$$

where $P_{c}$ and $P_{n}$ are the probabilities (0–1) of the current pixel and its neighboring pixel, and $w_{min}$ is set to $1\times 10^{-5}$, a small positive number to avoid errors while applying graph theory. Consequently, we added an additional column of nodes on both sides of the images and assigned them a maximum probability of 1. Doing this allows users to have the start and end nodes fixed on the top left and bottom right of images with the newly added columns, and the algorithm is able to traverse in the vertical direction of these columns with minimal resistance until the end node of the particular layer creates an even lower cost path.

**Figure 3:**
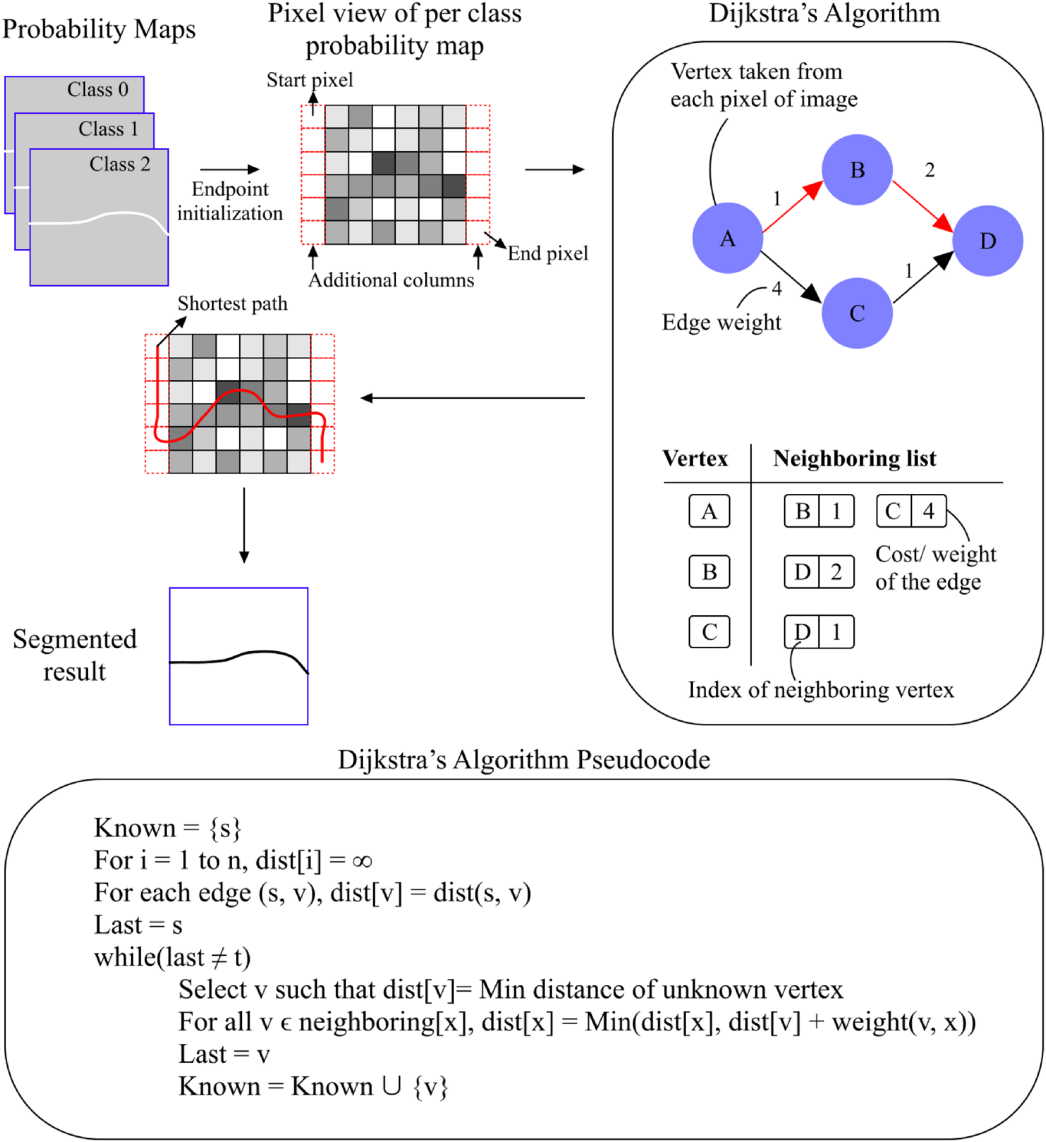
Overview of graph search application with automatic endpoint initialization on output probability maps of a sample of OCT image.

Moreover, to reduce intervention by users, we do not limit the segmented area between the top and bottom layer boundaries, nor limit the segmented direction. Every neighboring pixel (at most eight) is taken into consideration. When the directed weighted graph is found from the modified probability map, we created an adjacency list to represent this finite graph. Instead of using an adjacency matrix, an adjacency list is applied since the larger OCT image creates a larger graph with a larger number of nodes, therefore, using the adjacency matrix requires more memory storage and computation time^28^. In the adjacency list, each list describes a vertex and the set of its neighboring vertices with the assigned edge weight in the graph. By doing so, the connected vertex with the lower edge cost among all the neighboring pixels can be easily found based on the previously reported vertex. To delineate the shortest path, Dijkstra’s shortest path algorithm^29^, is implemented to traverse the whole image, without human interference, to find the minimum cost path. Once the image is segmented, the two additional columns can be removed, leaving an accurate cut and original size of the image for analysis preventing the error occurrence of endpoint initialization. The shortest path results, after removing the additional columns, are presented as the final predicted layer boundaries. The visualization of the graph search process is shown in Fig. 3.

### UNet (for comparative study)

To compare the performance of our proposed CNN-GS algorithm to a semantic image segmentation method, we employed a UNet architecture. UNet consists of an encoder and decoder linked together via skip connections. We utilized the UNet in^30^, but use padded 33$\times$33 convolutions instead of using unpadded convolutions in order to preserve spatial dimensions. For implementing CNN-GS and CNN-GS-skin, approximately 1200 images are sufficient to train the model, but for UNet, a larger number of training images are needed. To artificially increase the number of training examples, we augmented the data in the training set using random horizontal flips and elastic deformations at each iteration. Elastic deformations are achieved using a piecewise affine algorithm that places a 3$\times$3 grid of points on the image and randomly moves the neighborhood of these points around via affine transformations. These modifications are reasonable based on the physical properties of skin. The augmented images are then randomly cropped as 464$\times$256 pixel patches. This large patch size allows the network to capture context throughout the entire height of the image. A batch size of 4 is used.

To segment the epidermis, we labeled all pixels between Boundary 1 and Boundary 2 epidermis. Pixels above Boundary 1 are labeled as air, and pixels below Boundary 2 are labeled as tissue. To overcome the issue of heavily imbalanced classes and to force the model to learn boundaries, we used the weighted cross entropy loss function^30^. The model was optimized using a stochastic gradient descent optimizer with a high momentum of 0.99. The initial learning rate was set automatically using a learning rate finder algorithm^31^. The model was trained until the validation loss starts to plateau or degrade. The output of UNet is a pixel map containing raw, unnormalized scores for each class. We applied a softmax function on the scores to obtain the relative class membership probability. We then labeled pixels with over 50% probability of belonging to the epidermis class as epidermis. Finally, for each column, we labeled the top-most epidermis pixels as Boundary 1 and the bottom-most epidermis pixels as Boundary 2.

### Human study

Informed consent was obtained from all subjects and/or their legal guardian(s).

## Results and discussion

This section provides results from each stage of the CNN-GS methodology described in “Proposed framework”. The first part focuses on how the parameters of the proposed model structure were chosen and the performance of patch classification; the second part displays the outcome of the segmentation after applying graph search on probability maps generated by the trained model. Also included is a report on accuracy of ET thickness determinations using CNN-GS, CNN-GS-skin, and UNet for a validation set of OCT images (not used in training the models).

### CNN configuration optimization

The computational time for predicting ET across a single image (B-scan) using CNN-GS was found to be 60 s. The motivation for developing CNN-GS-skin was to reduce the execution time to less than 1 second while preserving the accuracy as much as possible; we allowed for a 3% decrease in accuracy. To achieve this goal, we developed a revised model with fewer parameters and implemented additional processes described below. The generated probability maps from our revised trained model were the intermediate results, therefore some error ranges were acceptable. Using this standard, we gradually adjusted the hyperparameters and implemented additional processes to obtain CNN-GS-skin (the final proposed model).

The model was tuned with a batch size of 512 and selecting optimum results. We used categorical cross-entropy and RMSprop optimizer^32^ for our model. All the models were trained identically as described in Stage 1 of “Proposed framework”. The model was trained until the validation loss started to plateau or degrade. The degradation rate was measured empirically. Increasing epochs and batch sizes did not significantly reduce the training loss, but increased the training computation and memory cost. In addition, to avoid model overfitting to the patchset, dropout^33^ was set in the model where a random number of nodes in the layer were ignored in each epoch, and early stopping was used to measure where to stop training.

#### Parameter tuning

The best configuration of the model depends on several factors including chosen dataset, model structure, and specific hyperparameters. CNN model structure contains various tunable hyperparameters^23^, such as filter and kernel size and number, pooling size, and type. To demonstrate the effects of different hyperparameters, in addition to comparing classification accuracy, we also compared the average execution time on target images from our model to the results taken from the baseline (CNN-GS) model. The execution time is calculated using the target patchset. The sections below show the results that have significantly influenced the model structure. We followed a sequential optimization where one parameter is modified at a time, and the remaining model settings stay constant. Once a better parameter set is selected, we updated our previous setup and tuned the next parameter, repeating until the last chosen parameter is tuned.

**Table 1:** Effect of modifying parameters of CNN-GS model for use to identify epidermal boundaries.

Effect of modifying parameters	CNN-GS (baseline)	Modified conv. layer filter	Modified filter count	Modified pooling kernel size	Modified ReLUs of FC layers	Reduction ReLUs of FC layers (i)	Reduction ReLUs of FC layers (ii)
Conv layer filter	$5 \times 5$	${\textbf{3}}\times {\textbf{3}}$	$3\times 3$	$3\times 3$	${\textbf{3}}\times {\textbf{3}}$	$3\times 3$	$3\times 3$
Filter count*	32, 32, 64	32, 32, 64	**8, 16, 16**	8, 16, 16	**8, 16, 16**	8, 16, 16	8, 16, 16
Pooling Kernel size*	$2\times 2,3\times 3,3\times 3$	$2\times 2,3\times 3,3\times 3$	$2\times 2,3\times 3,3\times 3$	${\textbf{2}}\times {\textbf{2}}, {\textbf{2}}\times {\textbf{2}}, {\textbf{2}}\times {\textbf{2}}$	${\textbf{2}}\times {\textbf{2}},{\textbf{2}}\times {\textbf{2}},{\textbf{2}}\times {\textbf{2}}$	$2\times 2,2\times 2,2\times 2$	$2\times 2,2\times 2,2\times 2$
ReLus of FC layers	64	64	64	64	**32**	16	8
Training accuracy	97.27%	96.48%	94.75%	95.40%	94.74%	94.36%	93.80%
Validation accuracy	96.68%	96.00%	94.63%	95.12%	94.50%	94.16%	93.62%
Testing accuracy	96.68%	96.10%	94.63%	95.23%	94.68%	94.86%	94.20%
# Parameters	114,916	93,924	20,276	29,492	16,532	10,052	6812
Execution time	60 s	56 s	10 s	13 s	9 s	9 s	9 s

Bolded parameters are those adopted for the remainder of the analyses.

*Conv* convolution, *ReLUs* rectified linear unit, *FC* fully connected.

^∗^Representation for each layer (Convolution 1, Convolution 2, Convolution 3).

**Convolutional layer filter size :** Each convolutional layer is performed on the input data with the use of filters. The filters are the feature detectors, where each filter convolves through the entire input and generates one feature map accordingly. The output size of the feature map is a result of the filter size. The results of reducing the filter size from 5$\times$5 in the baseline model (CNN-GS) to 3$\times$3 in all three convolutional layers are shown in Table 1. This change reduced the number of parameters somewhat, and slightly improved the execution time.

**Filter count:** After the selection of filter size, we considered the filter count parameter. The more filters, intuitively, the more explanatory factors are found, and the more the network learns, but learning a particular system may not require a large number of features: the most suitable number generally can be learned from experience. The experimental results are shown in Table 1. Reducing the filter number sacrifices some features extracted from the input, however, it reduces the number of parameters massively and reduces the amount of computation time needed for the following convolution. We selected the result, filter count = (8, 16, 16), because, with a relatively large training set, we did not want to lose too much information from the input. When filter count is (8, 16, 16), it has already eliminated about 78% of the parameters and deceased execution time by a factor of 5, with < 2% loss of accuracy.

**Pooling kernal size:** The function of pooling is to continuously reduce the input dimensionality leading to a smaller number of parameters and computations in the network. The maximum pooling layer downsamples the input by keeping the maximum activation in a given window. For feature extraction, we decided to utilize a larger filter size in the convolutional layer to explore sensible features and a small pooling kernel size to prevent useful information from being removed. Since we have already significantly decreased the number of parameters in convolutional layers, to not lose valuable data, we decreased the pooling kernel size. As can be seen in Table 1, the smaller the kernel size the model has, the larger the number of parameters it must learn from, leading to a longer training time in each epoch. However, it also leads to higher accuracy. Considering the trade-off between the additional training cost and the potential loss of valuable information, we opted for smaller size filters and a slight increase in execution time.

**Units for the fully connected (FC) layer:** Unlike convolutional layers, FC layers do not share parameters. On the contrary, they connect to every node from the previous layer, creating the majority of parameters inside the model, among all layers. Thereby, applying dropout to eliminate partial numbers, and controlling the unit value of FC layers can greatly reduce the number of parameters in the model. While CNN-GS models utilize two FC layers, only the unit value in the first FC layer is tuned, since the unit value of the final FC layer, the output layer, is used to reduce output to a single vector of probability (to identify whether the pixel is a boundary pixel or not). Table 1 shows that modifying the unit values can decrease parameters, as predicted by theory, and results in reduced training and execution time. The smaller rectified linear unit values (16, 8) lower the classification accuracy and number of parameters, but not the execution time. For future experiments, we selected a unit value of 32, as it has less than a 1% accuracy difference but reduces the number of parameters in the model by about 50%. In summary, we have demonstrated how simply tuning the parameters of the CNN-GS model enables a reduction in number of parameters and execution times. This improvement, while significant, is not sufficient to reach our goal of < 1 s, so we will explore further modifications to the model.

#### Loss function optimization

While training machine learning models, optimization algorithms are used to change attributes of the model, such as learning rate, and weights, to reduce losses and get the best possible results. The current error of the model has to be estimated repeatedly so that the weights can be updated to improve model learning and move to the next evaluation. This process requires loss functions. The choice of a suitable loss function depends on the predictive modeling problem. Our model is undertaking a multiclass classification where each patch is assigned to one of three classes, therefore, we compared the effect of applying two different loss functions commonly used for multiclass classifications. To perform the optimizations up to this point, we used the well known loss function ‘categorical_crossentropy’. To probe our choice of loss function, we reran the analysis using ‘Kull_Leibler_divergence’ (KL divergence). Cross entropy and KL divergence both measure the difference between two probability distributions, but cross entropy evaluates the number of bits needed to encode events from one distribution using the optimal code for another distribution, and KL divergence measures the information loss when one distribution is used to approximate another. We compared the use of cross entropy and KL-divergence on the previously identified best parameters from Table 1, and found the two tests had the same training time/epoch (115 s), and execution time (9 s). The training performance of these two loss functions is very similar, but cross entropy had slightly higher training, validation, and test accuracy (0.10–0.24% improvement) so we stayed with the selection of ‘categorical_crossentropy’ for our model.

#### Pruning and quantization

When optimizing a classification problem, it is always valuable to test different compression methods to see if they can reduce model complexity and computational cost without sacrificing test accuracy or execution speed. Network pruning focuses on reducing redundant weights or parameters, which are not sensitive to performance in a dense model. Pruning can lead to net reduction in inference time, but the degree to which it reduces time can vary widely depending on a specific system’s parameters. Network quantization compresses the original network by reducing the number of bits to represent model weights. By doing so, the weights can be quantized to small bits and the size of the model can be significantly reduced^34^. The results shown in Table 2 demonstrate that the original model without pruning or quantization shows slightly better results and shorter execution times. The fact that pruning did not make a significant difference suggests the model is already close to the minimal number of parameters needed to solve the task. Surprisingly, the quantized model has the longest execution time. This may be due to our hardware system, which cannot operate quantized data natively as is required for deep learning inference.

**Table 2:** Comparison of loss function applied in the model.

	Model w/pruning	Model w/quantization	Model w/o^∗^ Prun./quant.^∗^
Training acc	93.95%	94.56%	**94.74%**
Validation acc.	93.70%	94.40%	**94.50%**
Test acc.	93.77%	94.5%	**94.68%**
Predicting time	13 s	21 s	**9 s**

The selected parameter is labeled in bold.

^∗^The tuned model is based on results in Table 1.

#### Pixel skipping

**Figure 4:**
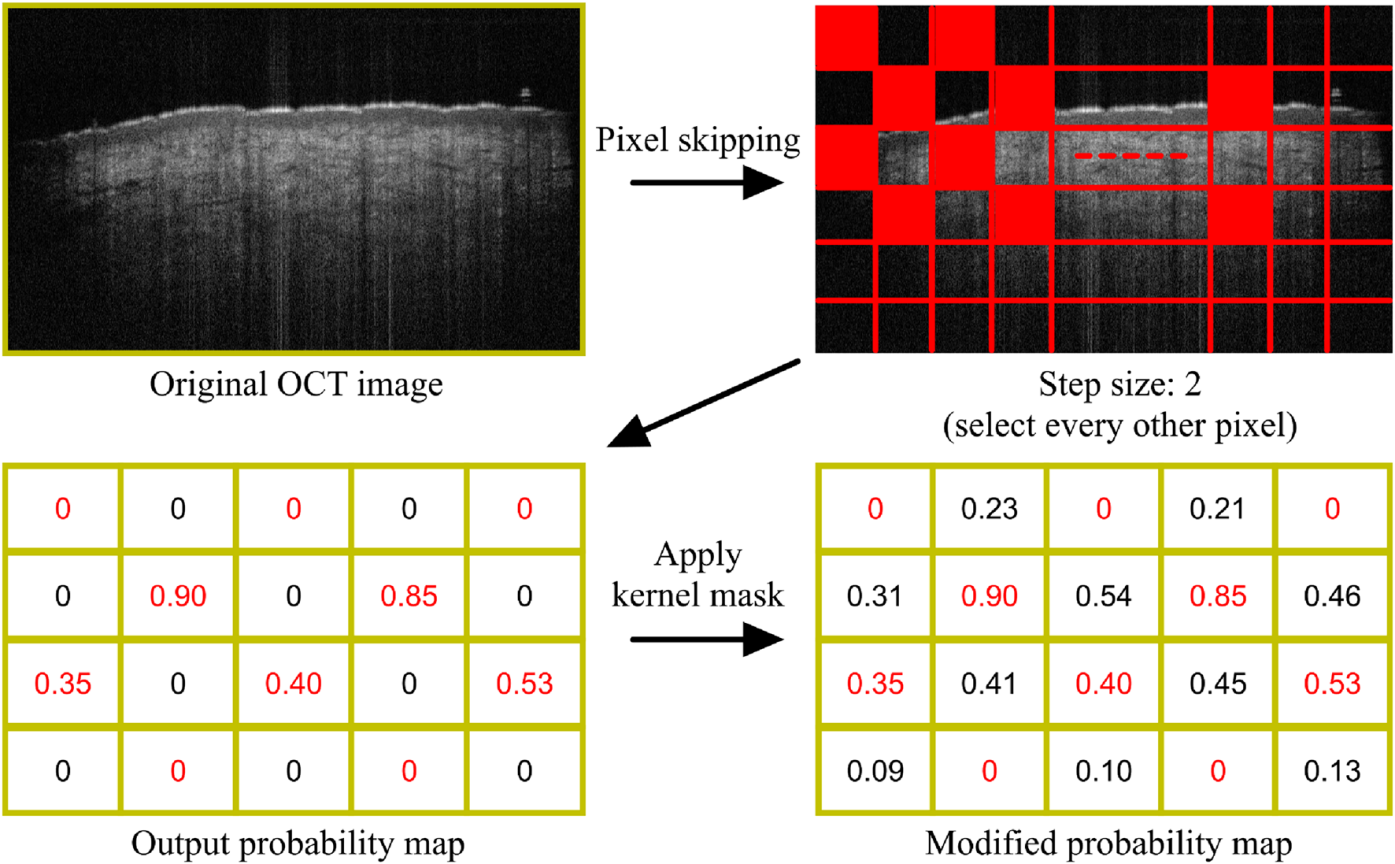
The scheme of pixel skipping. The red values in the output probability map are the predicted results of selected pixels from the CNN model. Next, the kernel is applied to calculate the mean of the sum of its neighboring pixels’ probability.

Up to this point, we simply optimized hyperparameters on the CNN-GS method and tested variations in the loss function and pruning and quantization to reduce execution time. However, at 9 s, we were nowhere near our goal of CPU analysis in under 1 s. We reflected that reducing the input size of the model needed for predicting can be used to save computation costs and reduce execution time. To preserve the predicted results, instead of directly decreasing the number of predicted patches, we implemented a simple technique called pixel skipping. Pixel skipping takes a pixel from the image and skips several pixels in between to get the next pixel, repeating until the last pixel in the image. Pixel skipping may be a very effective technique for analyzing the epidermis where the main outputs of interest are the boundaries of the epidermis. The effect of pixel skipping is to create a smaller size target patchset where patches are centered on selected pixels. After the model has predicted these selected pixels, we introduce a $kernel\;mask = [0 \hspace{0.6mm} 1 \hspace{0.6mm} 0, 1\hspace{0.6mm} 0\hspace{0.6mm} 1, 0 \hspace{0.6mm}1 \hspace{0.6mm}0]$ to calculate the mean of the sum of its neighboring pixels’ probability. If the probability is low in all of the pixels, it continues skipping. Figure 4 shows a simple example of applying pixel skipping on an OCT image.

**Figure 5:**
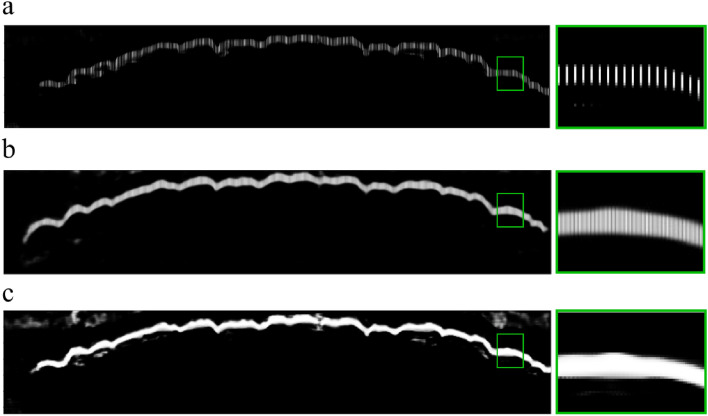
Visualization of results (**a**) with pixel skipping (step size = 4), (**b**) results after applying kernel matrix, and (**c**) without pixel skipping.

Figure 5 illustrates the pixel skipping concept. In Fig. 5a, the results were predicted on the pixel skip patchset only. In Fig. 5b, we display the results after putting the missing pixels back with the kernel mask, creating a more saturated image, and Fig. 5c shows the original results, without pixel skipping. With a step size equal to 2, which takes one pixel and skips the next pixel, the full target patchset is reduced in size to half. And when the step size is 4, the size reduces to a quarter. We then tested these three patchsets with our selected configuration and found no loss of accuracy for boundary selection. The results are presented in Table 3. The smaller patchset size has improved the execution time significantly.

**Table 3:** Predicting average processing time with patchset size.

Patchset size	Step size	Model w/o multiprocessing	Model w multiprocessing
Full target patchset	Full	9 s	1.224 s
Half	2	5 s	0.834 s
**Quarter**	**4**	2 s	**0.453 s**

Selected step size: 4 with multiprocessing where the model achieved execution average processing time of less than 1 s.

^∗^The model is based on use of optimized parameters from Table 1.

Significant values are in bold.

#### Effect of parallel processing on execution time

To further reduce the execution time, we used parallel processing on a multicore CPU. We noticed that the Python language predates multicore CPUs, and has a global interpreter lock (GIL), meaning only one thread can be executed at a time. As Python does not use multicore natively, it is a performance bottleneck in CPU-bound solutions. We designed a workaround using multiprocessing, which utilizes all possible CPU cores on users’ setup and handles several tasks in parallel. We created multiple processes, and each process is loaded with a trained model and chunks of the patchset to achieve parallelism. At first, multiprocessing did not significantly speed up the process. This is because creating copies of the trained model and needing to load the deep learning model setup on every processor resulted in increased inference time, causing the system to slow down. After some experimentation, we found that the installed Tensorflow-CPU version affects the inference time, therefore, Tensorflow-CPU versions greater than 2.3 must be used for speeding up. Our hardware and OS configuration was: Intel(R) Xeon(R) Gold 624R CPU @ 3.00GHz $\times$ 8664 (and DDR5 6000MHz RAM) with Tensorflow-CPU version 2.9.4 and Keras version 2.4.3. With this setup, we reran the model using our three sizes of target patchsets. From Table 3, a smaller size input patchset decreased the execution time, and the patchsets processed with multiprocessing show improved execution time.

**Table 4:** CNN structure comparison of CNN-GS and CNN-GS-skin.

	CNN-GS*^∗^	CNN-GS-skin
Conv1 + ReLU	(5 $\times$ 5), 32 − 1	(3 $\times$ 3), 8 − 1
Max pooling	(2 $\times$ 2) − 2	(2 $\times$ 2) − 2
Conv2 + ReLU	(5 $\times$ 5), 32 − 1	(3 $\times$ 3), 16 − 1
Avg. pooling	(3 $\times$ 3) − 2	(2 $\times$ 2) − 2
Conv3 + ReLU	(5 $\times$ 5), 64 − 1	(3 $\times$ 3), 16 − 1
Avg. pooling	(3 $\times$ 3) − 2	(2 $\times$ 2) − 2
FC Layer1 + ReLU	64	32
Dropout	0.1	0.1
FC Layer 2 + Softmax	4	4
# of param.	114,916	16,532
Training acc.	97.27%	94.74%
Validation acc.	96.73%	94.50%
Test acc.	96.68%	94.68%
Execution time	60 s	<0.5 s

Representation of number: for convolution layers, (kernel size), filter count—strides; for pooling layers, (kernel size)—strides.

^∗^Parameters proposed by Fang et al.^20^.

In summary, based on the experimental discoveries presented in “Parameter tuning”, “Loss function optimization”, “Pruning and quantization” and “Pixel skipping”, a tuned CNN configuration was achieved. An overview of the baseline (CNN-GS) and optimized (CNN-GS-skin) configurations are depicted in Table 4. The size of filters in convolutional layers, as well as pooling kernel size, were modified. Fewer filters and fewer units of FC layer were also used. Besides refinement of the model, reduction of the patchset size via pixel skipping and multiprocessing was also implemented. In addition to the analytical information in Tables 1, 2 and 3, where the accuracy is analyzed patch by patch, we provide below additional data below to support our model performance.

**Figure 6:**
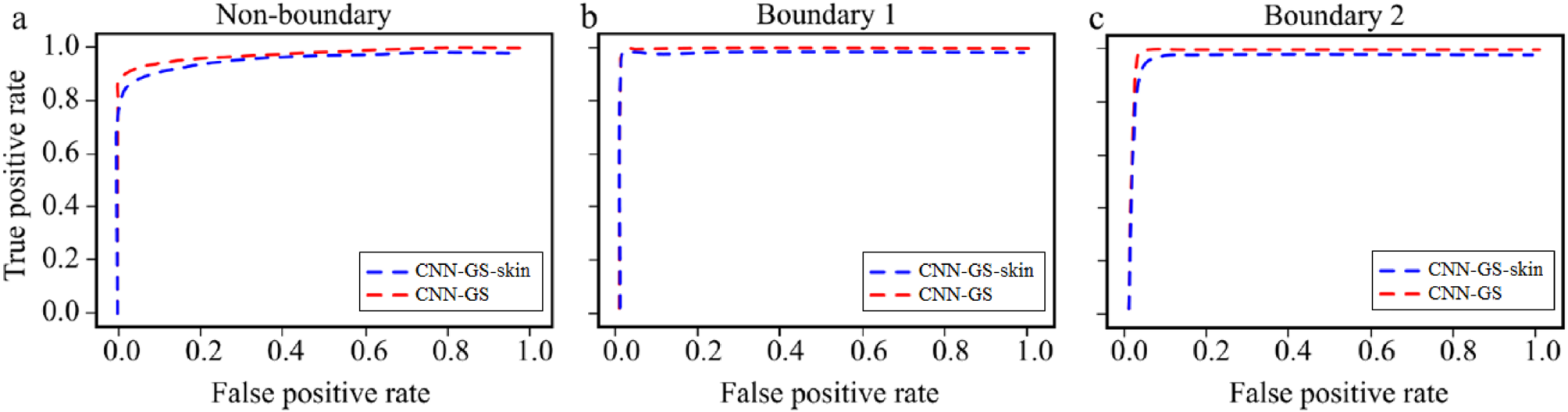
ROC curve of CNN-GS and CNN-GS-skin for (**a**) non-boundary (epidermis), (**b**) boundary 1 (SC), and (**c**) boundary 2 (DEJ).

The receiver operating characteristic (ROC) demonstrates how well a model can distinguish between classes. The higher the area under the curve (AUC), the better the model. The ROC curves in Fig. 6 were plotted for the SC (boundary 1), the epidermis (non-boundary), and the DEJ (boundary 2). The numerical measurements are presented in Table 5. We can see that numerical results of the CNN-GS-skin model have minor differences compared to the CNN-GS model, however, these differences cause no significant reduction in accuracy. CNN-GS-skin has promising results and high efficiency. It has shrunk about 90% of the parameters but kept an average 94.68% testing accuracy, only $\sim$2% less than CNN-GS. More importantly, the proposed model runs, in the test phase, in less than 0.5 s.

**Table 5:** Classification report of CNN-GS and CNN-GS-skin.

	CNN-GS				CNN-GS-skin			
	PPV	Sensitivity	F1	AUC	PPV	Sensitivity	F1	AUC
Non-boundary	0.97	0.89	0.93	0.98	0.95	0.82	0.88	0.97
Boundary 1	0.98	1.00	0.99	1.00	0.98	0.99	0.98	1.00
Boundary 2	0.93	0.98	0.95	0.99	0.88	0.96	0.92	0.99

*PPV* positive predictive value, *F1* F score measure of accuracy, *AUC* area under the receiver operating characteristic curve.

### Segmentation performance analysis

**Figure 7:**
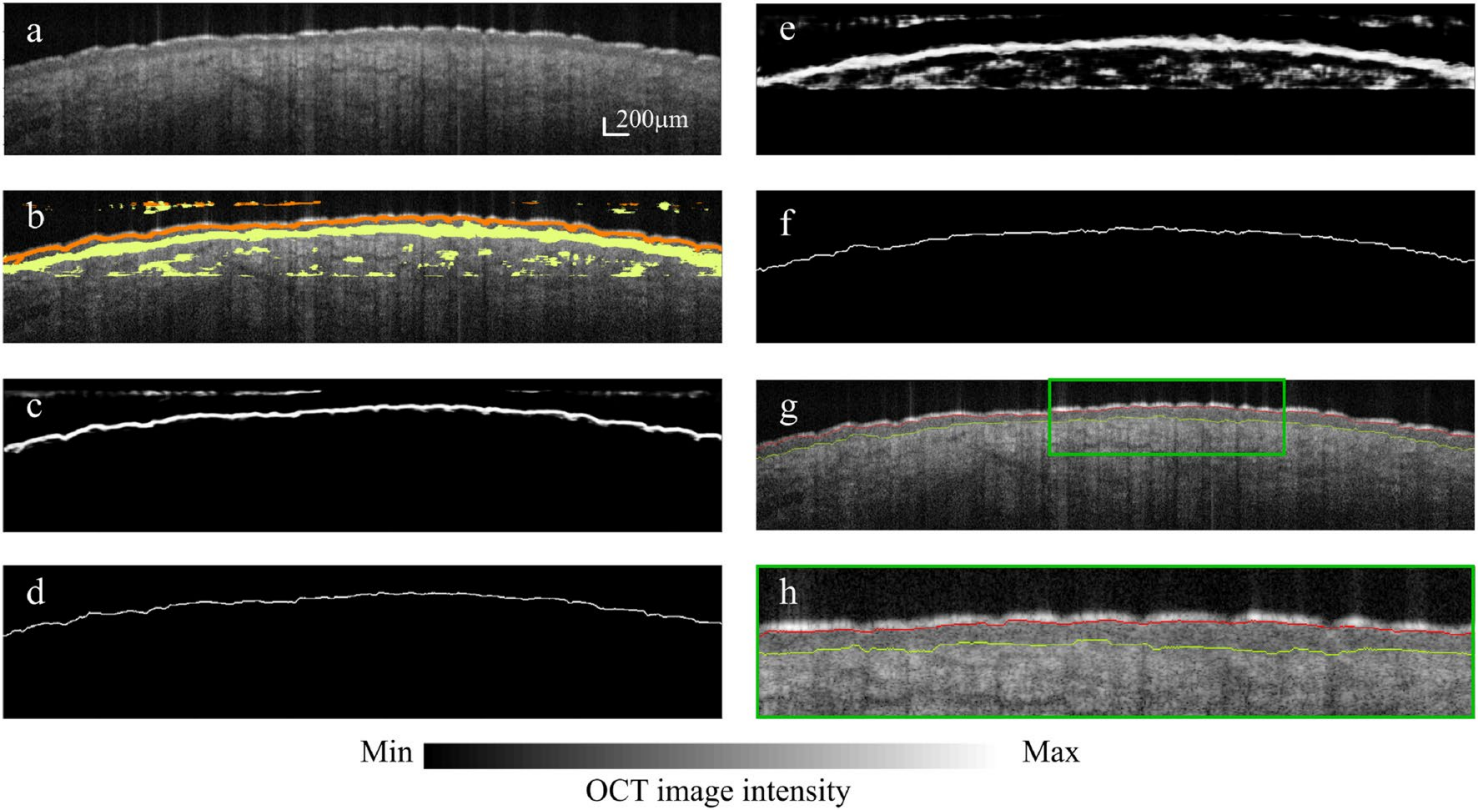
Segmentation results of CNN-GS implementation on skin OCT image. (**a**) Sample skin OCT image. Scale bar, 200 $\upmu$m. (**b**) Intermediate results generated by the proposed model. (**c**) Patch classification and (**d**) layer segmentation on boundary 1. (**e**) Patch classification and (**f**) layer segmentation on Boundary 2. (**g**) Final segmentation of target image. (**h**) Magnified segmentation of (**g**) $\sim$3$\times$ magnification.

Previously, accuracy was determined for each analyzed patch. In this section, the patch results are converted into computed segmentation performance, and the computed segmentation performance for the different frameworks are analyzed and compared with the manual segmentation ground truth. The boundaries are delineated through the procedure described in stage 2 of the CNN-GS framework. The visual results of the CNN-GS methodology overlaid on the target image are illustrated in Fig. 7. To analyze the predicted segmentation, we took manual segmentation as the gold standard and calculated the similarity between predicted segmentation and manual segmentation. Note that, manual segmentation is a somewhat subjective process (results of manual segmentation varied by 0–4 pixels among the 4 independent analysts, and the ground truth was defined as the median result for each pixel). For each boundary in the OCT image, we marked the position for both predicted and manual segmentation and calculated the mean error in terms of pixel size. After taking the value of these differences, the mean difference and standard deviation were calculated. The position accuracy (3) was also measured based on the mean of the number of boundary positions that were predicted correctly and the total number of labeled pixels. Please note: axial pixel size $\approx$ 3.25 $\upmu$m and system axial resolution is 10 $\upmu$m. To correct human bias in manual segmentation, we calculated the visual error tolerance, indicating the acceptable error ranges for humans while analyzing the OCT boundaries. For this experiment, we shifted boundaries by 1–4 pixels and checked the results with seven experts, who are familiar with OCT imaging, by asking them to compare the modified image to their results. Their responses indicated predicted positions are generally satisfactory if within 2 pixels (2).

2 $$\begin{aligned} Correct\;Position = |P_{pred} - P_{man}|\le 2 \end{aligned}$$

3 $$\begin{aligned} Position\;Accuracy = \frac{\#\;of\;Correct\;Position}{Total\;Labeled\;Pixels} \end{aligned}$$

where $P_{pred}$ is position index of current predicted pixel; and $P_{man}$ is the corresponding manual segmented pixel position.

**Figure 8:**
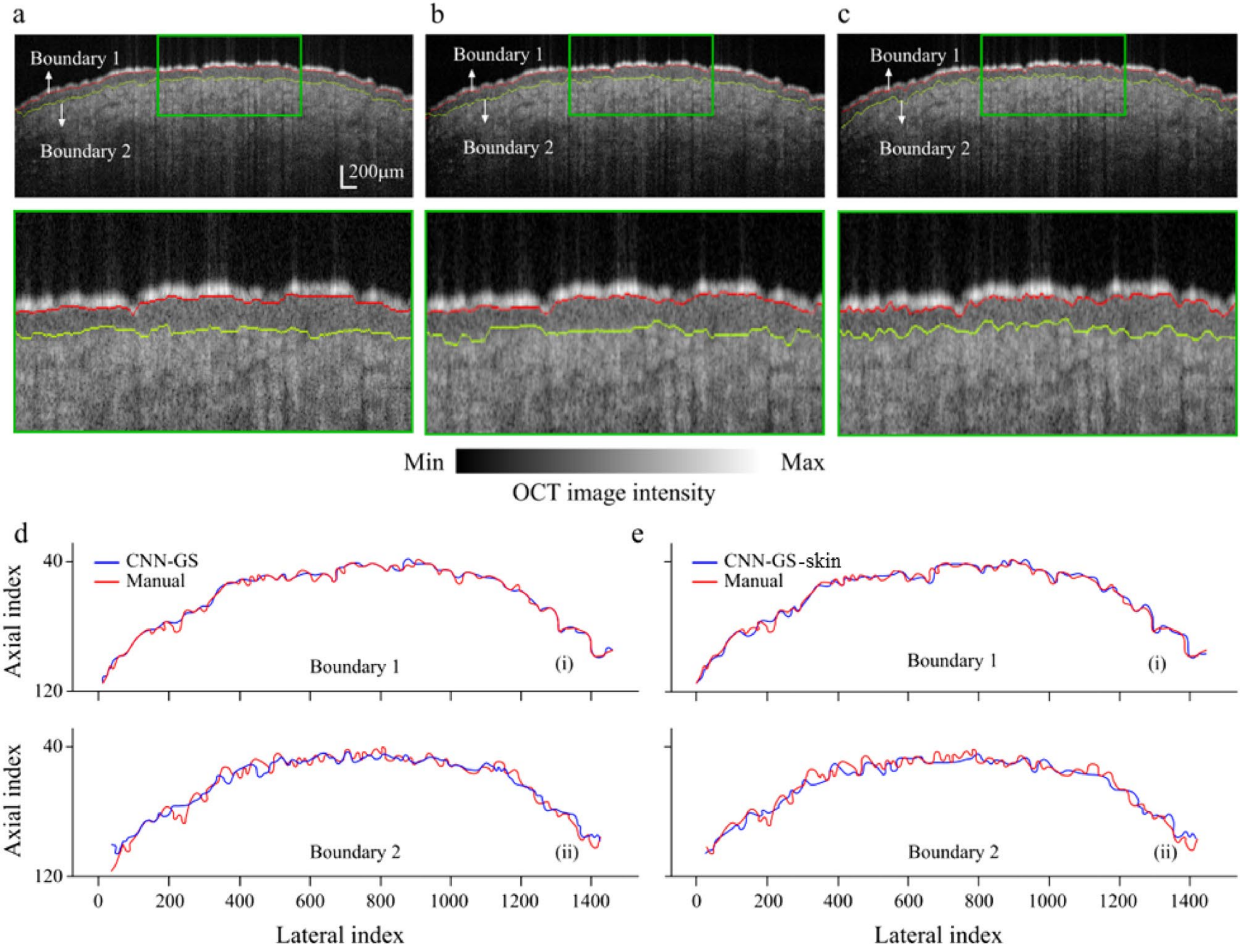
Visual comparisons between (**a**) (i) CNN-GS (ii) $\sim$3$\times$ magnification, (**b**) (i) CNN-GS-skin (ii) $\sim$3$\times$ magnification, and (**c**) (i) manual segmentation (ii) $\sim$3$\times$ magnification. Contour of boundaries for (**d**) CNN-GS and (**e**) CNN-GS-skin versus manual segmentation.

**Table 6:** Border position error (in pixels) comparing CNN-GS and CNN-GS-skin with manual segmentation, for each layer boundary in skin OCT images.

	Comparison with manual segmentation					
	CNN-GS			CNN-GS-skin		
	Mean difference	Standard deviation	Position accuracy	Mean difference	Standard deviation	Position accuracy
Boundary 1	0.02	0.14	98.00%	0.02	0.15	97.27%
Boundary 2	0.05	0.24	75.44%	0.06	0.25	74.40%
Overall	0.08	0.28	86.36%	0.09	0.30	85.81%

Results are displayed as mean difference, standard deviation, and position accuracy. 1 pixel $\approx$ 3.25 $\upmu$m.

To determine the ability of CNN-GS-skin, besides comparing it to manual segmentation, we compared it to CNN-GS to check accuracy. As the results show in Table 6, for Boundary 1 (the SC) position accuracy is very high for both CNN-GS and CNN-GS-skin. For Boundary 2 (the DEJ), both models performed less well. And, in fact, Boundary 2 is not instinctively visible for people to delineate: when we combined several annotations for the same image, the results still fluctuated. However, even though CNN-GS-skin performs worse than CNN-GS during patch classification, the results show a negligible difference in boundary delineation (overall less than 1% reduction in accuracy). This supports our intuition that the system could tolerate a 2–3% decrease in patch classification accuracy without significantly reducing ET boundary detection. To demonstrate this conclusion, the segmented images using both models and manual segmentation are shown in Fig. 8. Also plotted are the contours of the two boundaries to show how similar our predicted results (CNN-GS-skin) are to the prediction using the CNN-GS and the manual segmentation. As is shown, the second boundary is more divergent compared to the first boundary, which results in less position accuracy, but still within the tolerance of manual annotation.

To demonstrate the computational strength of our CNN-GS approach, we compared our results to a method run by UNet, an encoder-decoder architecture that eliminates all pre-processing procedures on patch creation. UNet is a more complicated model that is typically trained on GPU; however, using the trained UNet, introduced in 2.4, we processed our testing images on CPU and obtained an execution time of 27.57 s with a standard deviation of 0.1522 s; the execution time may be improved by incorporating some speed-up algorithms. In terms of execution time, CNN-GS-skin (< 0.5 s) far outperforms UNet. To evaluate the accuracy of the ET measurement, we defined the parameter “ET Accuracy” as shown in Eq. (4).

4 $$\begin{aligned} ET Accuracy = \frac{\#\;of\;Img_{correct}}{\#\;of\;Test\;Images} \end{aligned}$$

where $Img_{correct}$ represents those predicted OCT images having the ET within standard deviation compared to manual segmentation.

**Table 7:** Comparison with manual segmentation of position accuracy (Acc.) at stratum corneum (SC) and dermal epidermal junction (DEJ) and epidermal thickness (ET) accuracy.

	CNN GS vs. manual segmentation			CNN-GS-skin vs. manual segmentation			UNet vs. manual segmentation		
	Position acc. (%)		ET acc. (%)	Position acc. (%)		ET acc. (%)	Position acc. (%)		ET acc. (%)
	SC	DEJ		SC	DEJ		SC	DEJ	
Forearm	95.15	84.70	95.3	93.00	80.47	94.01	94.34	87.12	93.11
Back of hand	99.10	86.15	94.82	98.8	88.15	92.48	98.37	85.74	91.21
Forehead	99.32	85.31	94.42	98.62	84.81	93.36	97.43	84.81	93.79
Neck	99.50	91.24	98.41	98.5	82.20	94.56	96.62	90.43	95.17
Palm	99.63	97.54	98.96	99.63	97.04	99.96	98.34	96.87	96.54
Overall	98.54	88.98	96.38	97.71	86.53	94.87	97.63	87.19	98.26

**Figure 9:**
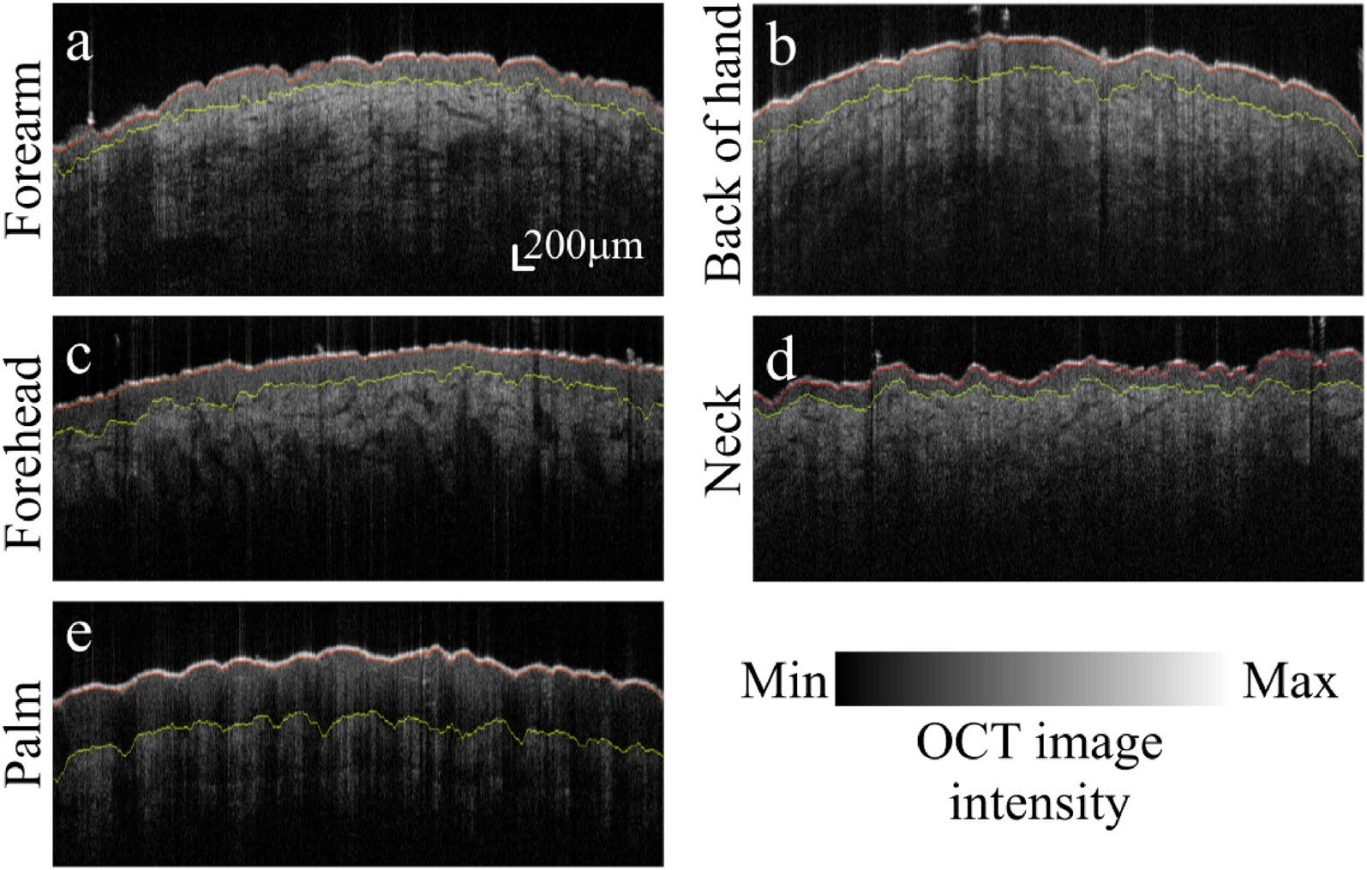
Application of CNN-GS-skin to various skin OCT images from five sample images. Original OCT image with segmented results of (**a**) forearm, (**b**) back of hand, (**c**) forehead, (**d**) neck, and (**e**) palm.

**Figure 10:**
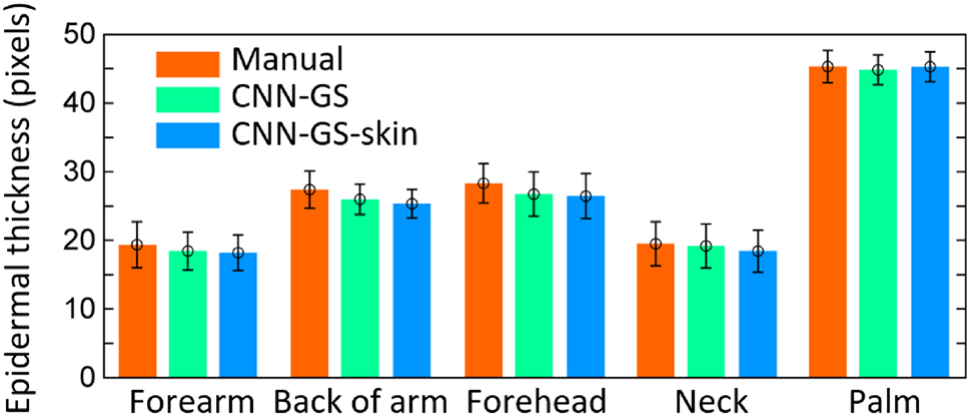
Comparison of epidermal thickness (in pixels) between CNN-GS using baseline and proposed model and manual segmentation (1 pixel $\approx$ 3.25 $\upmu$m) for five sample images.

The robustness and application of our algorithm on different body sites are visually demonstrated in Fig. 9. For detailed analysis, we calculated the position accuracy and epidermal thickness by body site in Table 7 where we compared CNN-GS-skin with CNN-GS and UNet to verify our methodology. Defining the performance of our algorithm as the absolute difference between the average value obtained from each model and manual segmentation and divided by the results of manual segmentation, we reached $\approx$95%(0.12) accuracy across all body sites (i.e., OCT images taken from the forearm, back of hand, forehead, neck, and palm of 63 healthy individuals). Additionally, in Fig. 10, the bar chart was plotted with the epidermal thickness and a standard deviation to show the comparison of predicted and manual segmentation, which further supports our proposed model having competitive performance. From these results, even though CNN-GS has slightly better performance, the average predicted epidermal thickness for each location is similar to the manually segmented thickness. We observed variability of ET measurement accuracy in different skin locations. We note that these values for epidermal thickness are well within published values for these body sites^35–37^.

## Conclusion

OCT is a three-dimensional high-resolution imaging modality that has been used in assessing a range of skin conditions including psoriasis, contact or atopic dermatitis, lichen planus, acne lesion^3,4^, papule^5^, wound healing^6^ and skin cancer^7^. In most skin diseases, there is an alteration in the epidermis, which may result in epidermal-dermal layer thickness change^9,38–46^. Because of high multiple scattering in skin, accurate annotation of epidermis structure based on OCT image is a challenging task^47^. Moreover, manual segmentations are extremely time-consuming with variable interpretation^48^, repeatability, and interobserver agreement, which is not suitable for clinical applications. Additionally, the traditional OCT epidermal segmentation that works based on the detection of the minimum local intensity of the DEJ highly depends on the image quality and the skin pathologies^49^. Therefore, automated segmentation using deep-learning methods has become increasingly popular in OCT imaging. Although many researchers have implemented deep convolutional neural networks and achieved great success in the segmentation tasks^5,47,50–52^, the execution time of these methods is too long, which limits their practicality^49,53,54^.

We have developed a method that is capable of accurately segmenting layer boundaries from different body sites in near real time, although there are enhancements that can be made to improve boundary delineation such as increasing the number of OCT images in our dataset, expanding number of manual segmentations to make our analysis more objective, model optimization to enhance classification ability, and also methods to reduce the complex process of patch-based encoder network. The CNN-GS-skin algorithm has shown a convincing ability to delineate boundaries while achieving high computational efficiency. We could have selected a more complex CNN or used higher-efficiency hardware to improve our performance, but we chose to reduce the cost of computation within specified limitations, requiring the implementation of additional techniques such as pixel skipping and multiprocessing. These add-ons significantly reduced the computational cost of our method. The reason we are interested in CPU is that most OCT imaging systems and their reconstruction algorithms are already implemented on CPU, rather than GPU. Developing a methodology for CPU is therefore highly preferable. There are some pitfalls that need to be addressed. A major issue is that this methodology is reliant on an annotated dataset from manual segmentation, which has biases and variability. The annotated data has not been verified by histology, but has been verified to be accurate within 2 pixels (6.5 $\upmu$m). Hence, the manual segmentation we took as the gold standard for analysis is also not perfect. In addition, our OCT image dataset may contain some imaging artifacts causing errors for segmentation. Despite the acknowledged shortcomings, this approach was validated on a variety of body regions, which is an important step to making this methodology accessible and practical in the clinic and assisting with an early-stage diagnosis of skin diseases.

Nevertheless, our proposed system stands out because of its simplicity, efficiency, and versatility, which makes it a robust method for automatic layer segmentation.

## Data Availability

The datasets used and/or analyzed during the current study and the CNN code are available from the corresponding author on reasonable request.
